# Biological Recovery of Platinum Complexes from Diluted Aqueous Streams by Axenic Cultures

**DOI:** 10.1371/journal.pone.0169093

**Published:** 2017-01-03

**Authors:** Synthia Maes, Ruben Props, Jeffrey P. Fitts, Rebecca De Smet, Frank Vanhaecke, Nico Boon, Tom Hennebel

**Affiliations:** 1 Center for Microbial Ecology and Technology (CMET), Department of Biochemical and Microbial Technology, Ghent University, Ghent, Belgium; 2 Department of Civil and Environmental Engineering, Princeton University, Princeton, NY, United States of America; 3 Department of Medical and Forensic Pathology, Ghent University, Ghent, Belgium; 4 Department of Analytical Chemistry, Ghent University, Ghent, Belgium; University of Edinburgh, UNITED KINGDOM

## Abstract

The widespread use of platinum in high-tech and catalytic applications has led to the production of diverse Pt loaded wastewaters. Effective recovery strategies are needed for the treatment of low concentrated waste streams to prevent pollution and to stimulate recovery of this precious resource. The biological recovery of five common environmental Pt-complexes was studied under acidic conditions; the chloro-complexes PtCl_4_^2-^ and PtCl_6_^2-^, the amine-complex Pt(NH_3_)_4_Cl_2_ and the pharmaceutical complexes cisplatin and carboplatin. Five bacterial species were screened on their platinum recovery potential; the Gram-negative species *Shewanella oneidensis* MR-1, *Cupriavidus metallidurans* CH34, *Geobacter metallireducens*, and *Pseudomonas stutzeri*, and the Gram-positive species *Bacillus toyonensis*. Overall, PtCl_4_^2-^ and PtCl_6_^2-^ were completely recovered by all bacterial species while only *S*. *oneidensis* and *C*. *metallidurans* were able to recover cisplatin quantitatively (99%), all in the presence of H_2_ as electron donor at pH 2. Carboplatin was only partly recovered (max. 25% at pH 7), whereas no recovery was observed in the case of the Pt-tetraamine complex. Transmission electron microscopy (TEM) revealed the presence of both intra- and extracellular platinum particles. Flow cytometry based microbial viability assessment demonstrated the decrease in number of intact bacterial cells during platinum reduction and indicated *C*. *metallidurans* to be the most resistant species. This study showed the effective and complete biological recovery of three common Pt-complexes, and estimated the fate and transport of the Pt-complexes in wastewater treatment plants and the natural environment.

## Introduction

The growing importance and use of platinum in clean and high-tech products in the last 30 years have induced the production of Pt loaded waste streams and the accumulation of platinum in the environment [1, 2]. For example, deterioration of automotive catalysts leads to the emission of Pt particles into the environment, part of which gets drained by stormwater into sewers [3]. Platinum is also the crucial building block of chemotherapeutic drugs such as cisplatin and carboplatin, and the excreted human metabolites contaminate both hospital and municipal wastewaters [1]. Finally, liquid waste streams (often diluted) containing platinum are also produced from the application of industrial catalysts, the manufacturing of jewelry and electronics, and both primary mining and precious metal recovery activities [2, 3].

The resulting residual platinum appears in different complexes in wastewater, with inorganic or organic ligands, such as cisplatin (*cis*-PtCl_2_[NH_3_]_2_), carboplatin (*cis*-(Pt[NH_3_]_2_[1,1-cyclobutanedicarboxylato])), and their metabolites, chloro-complexes Pt(II)Cl_4_^2-^ and Pt(IV)Cl_6_^2-^ or amine-complexes such as Pt(NH_3_)_4_Cl_2_, resulting from leaching or metal refinery processes [4–6]. The metal’s fate in a wastewater treatment plant or in the receiving environment depends largely on the metal’s speciation and the matrix composition of the waste stream [2]. An effective removal of the precious metal is advised to both lower the pollutant load in the environment, based on the pollution prevention principle, and since the behavior and impact of species such as cisplatin is mainly unknown in the environment [7]. Moreover, platinum’s high market value (av. 34.7 $ g^-1^ in 2015 [8]) and criticality stimulate the effective recovery and valorization of critical resources [9, 10]. Case by case, it should be questioned if the targeted Pt-complex could be removed and recovered from the waste stream, and whether this recovery could be interesting from an economical point of view [2].

Biotechnologies based on living biomass can serve as low-cost and green treatment techniques to recover platinum at low concentrations [11, 12]. The effective removal of platinum by different axenic cultures has been demonstrated before; PtCl_4_^2-^ and PtCl_6_^2-^ were sorbed by *Shewanella putrefaciens* [12], PtCl_6_^2-^ was reduced by *Shewanella algae* [13] and an undefined Pt-complex was reduced by *Cupriavidus metallidurans* [14]. However, the metal speciation can hamper an effective metal removal [2]. Complex waste streams such as highly acidic saline streams originating from metal refinery processes can be considered too challenging for conventional biological wastewater treatment plants (WWTP). They require specialized mixed cultures adapted to the prevalent conditions [11, 15].

The aim of this study was to further elaborate the biological recovery of different synthetic platinum complexes, representative for diluted Pt containing wastewaters of interest. It is important to explore the fate of these common Pt-complexes once they have entered a wastewater treatment plant or the environment. Therefore, this study investigates the relationship between Pt-speciation and the observed recovery by axenic cultures and the effect of the different Pt-complexes on the cell viability. The studied Pt-complexes include; chloro-complexes PtCl_4_^2-^ and PtCl_6_^2-^, present in e.g. run-off waters or industrial process streams, cisplatin, carboplatin, and a Pt-amine complex Pt(NH_3_)_4_Cl_2_, being found in metal refinery streams. A series of axenic bacterial species (*Shewanella oneidensis* MR-1, *Cupriavidus metallidurans* CH34, *Geobacter metallireducens*, *Bacillus toyonensis*, and *Pseudomonas stutzeri*), commonly present in wastewater plants treating metal contaminated effluents, was examined. In this research, it was evaluated which bacteria recovered platinum, under which conditions (with and without electron donor) and how this was affected by the metal speciation. The morphology and metal speciation of the precipitated Pt particles were investigated, as well as the viability of the axenic cultures during Pt recovery. The results of this study can be used as a prediction of the fate and transport of Pt salts in wastewater treatment plants.

## Materials and Methods

### Bacterial cultures and growth conditions

*Shewanella oneidensis* MR-1 was obtained from the BCCM/LMG Bacteria Collection (Gent, Belgium; LMG 19005) and *Cupriavidus metallidurans* CH34 was obtained from SCK•CEN (Mol, Belgium). Both species were grown aerobically in Lysogeny broth (LB-Lennox) medium overnight at 28°C. *Geobacter metallireducens* was obtained from the Deutsche Sammlung von Mikroorganismen und Zellkulturen GmbH (DSMZ; ATCC 53774) and was cultivated anaerobically in DSMZ medium 579 at 28°C for 7 days. *Bacillus toyonensis* and *Pseudomonas stutzeri* were isolated from a wastewater treatment plant which processes metal streams, and were grown aerobically in Lysogeny broth (LB-Lennox) medium for 48 h at 28°C.

### Platinum recovery experiments

The five different bacterial species were used to study the recovery of five different Pt-complexes; K_2_PtCl_4_ (Sigma-Aldrich, USA), K_2_PtCl_6_ (Sigma-Aldrich, USA), Pt(NH_3_)_4_.2HCO_3_ (Alfa Aesar, Germany), cisplatin (Alfa Aesar, Germany), and carboplatin (Alfa Aesar, Germany). Cells from the cultures *S*. *oneidensis*, *C*. *metallidurans*, *B*. *toyonensis*, and *P*. *stutzeri* were harvested by centrifugation (7000 g, 7 min) and were then washed twice with 25 mL phosphate buffer (8.5 g L^-1^ Na_2_HPO_4_.7H_2_O and 3 g L^-1^ KH_2_PO_4_). The washed cells were suspended in the phosphate buffer to a final optical density of 1 (OD_610nm_) and added to 120 mL glass serum bottles (50 mL cell suspensions). The glass bottles were flushed by 20 repeated cycles of N_2_ overpressure and vacuum underpressure. In the case H_2_ was used as electron donor, the headspace was replaced with 100% H_2_-gas. In the case of formate and acetate, 50 mM formate or 12.5 mM acetate was dosed to the biomass suspension. Subsequently, platinum was dosed to a final concentration of 100 mg L^-1^ Pt (2.5 g L^-1^ Pt 1 M HCl stock). The glass bottles were incubated and continuously mixed at 100 rpm and 28°C during the experiment.

The protocol was slightly modified for experiments based on *G*. *metallireducens*. The cells were centrifuged at 8000 g for 7 min, washed and suspended in 30 mM NaHCO_3_ to a final optical density of 0.31. All steps were executed in an anaerobic closet (37°C, 80% N_2_/20% CO_2_).

The washed biomass suspensions were all characterized by pH 7.0–7.1 prior to Pt dosage. The pH was adjusted to pH 1.8–2.2 at the start of the experiment. Samples were analyzed by inductively coupled plasma optical emission spectrometry (ICP-OES) and Pt recovery efficiencies were calculated after 48 h.

### Inductively coupled plasma optical emission spectrometry (ICP-OES)

Experimental details on ICP-OES analysis were previously described by Maes et al. [11]. The platinum concentrations were determined with a Spectro Arcos ICP-OES (Spectro Analytical Instruments GmbH, Kleve, Germany).

### X-ray absorption spectroscopy

Biomass pellet samples of cultures *S*. *oneidensis*, *C*. *metallidurans* and *G*. *metallireducens* were investigated by X-ray absorption spectroscopy after the recovery of the platinum chloro-complexes Pt(II)Cl_4_^2-^ and Pt(IV)Cl_6_^2-^. The indication “aerobic” or “anaerobic” refers to the atmospheric condition during cultivation. Anaerobic *S*. *oneidensis* was cultivated according to Schuetz et al. [16] with 50 mM ferric citrate as terminal electron acceptor. All recovery experiments were executed under anaerobic conditions. H_2_-gas was always applied as electron donor, except for the aerobic *S*. *oneidensis* sample with Pt(IV)Cl_6_^2-^ (with formate).

Experimental details on X-ray absorption spectroscopy were previously described by Maes et al. [11]. All μXAS spectroscopy measurements were performed using the microprobe beamline X27A at the National Synchrotron Light Source (NSLS), Upton, NY. Aliquots of fully hydrated Pt-biomass samples were transferred to an air-tight polypropylene bag to prevent drying. Samples were secured to an x, y, z motorized stage 45 degrees to the incident beam and 13-element HGe Canberra fluorescence detector. The beam spot-size on the sample was maintained at ca. 15 μm.

By means of X-ray Absorption Near Edge Structure (XANES) and Extended X-ray Absorption Fine Structure (EXAFS) spectroscopies, the oxidation state and first-shell coordination environment of the Pt phase associated with the biomass was examined. All spectra were collected at room temperature. X-ray fluorescence was measured from 150 eV below to 800 eV above the Pt L3-edge. Absolute x-ray energy calibration was based on the first inflection point of standard Pt (11 919 eV) metal foil, which was collected in transmission mode as an internal calibration during each scan. Normalization, calibration and averaging of the XAS spectra and *ab initio* fitting of the EXAFS region of the spectra were performed using Athena and Artemis software [17].

### Transmission electron microscopy (TEM)

After the recovery experiments were finished, the bacterial suspensions were washed twice with the according washing buffer; 30 mM NaHCO_3_ for *G*. *metallireducens*, phosphate buffer for all other species. Samples were stored overnight and the supernatants were removed. The TEM analysis was then performed as previously described by Maes et al. [11], using a Zeiss TEM900 transmission electron microscope (Carl Zeiss, Oberkochen, Germany).

### Cell viability by flow cytometry analysis

Partial viability of the strains was assessed during recovery experiments by dual stain flow cytometry as described elsewhere [18]. Briefly, bacterial cells were stained with a mixed SYBR^®^ Green I (SG, 10 000x concentrate, Invitrogen) and propidium iodide (PI, 20 μM, Invitrogen) staining solution which resolves membrane damaged from intact bacterial cells. At the start of the recovery experiments the suspension was acidified to pH 2 and the studied Pt-complex and H_2_-gas were added. Samples taken during batch experiments were diluted immediately 100 times in sterile, 0.22 μm filtered phosphate buffer and stored at +4°C until further analysis. Prior to analysis, samples were if necessary further diluted to approximately 10^6^ cells mL^-1^ and stained with 10 μL mL^-1^ of the staining solution (final concentration of 1x SG and 4 μM PI). The stained samples were incubated for 20 minutes in the dark at 37°C and immediately analyzed on a BD FACSVerse (BD Biosciences, Erembodgem, Belgium) equipped with a 20 mW 488 nm blue laser, 40 mW 405 nm violet laser and a 640 nm red laser. Green and red fluorescence intensities corresponding to respectively the SG and PI emission wavelengths were collected through a 527 ± 32 nm band pass and 700 ± 54 nm band pass filter. All samples were collected and analyzed in triplicate within 24 hours of sampling. Cell counts were extracted from manually drawn gates on the green vs. red fluorescence intensity plots as described elsewhere [19]. The limit of detection was 33.3 x 10^3^ cells mL^-1^.

## Results and Discussion

### Recovery of platinum complexes by axenic cultures

The recovery of five different platinum complexes was tested using five axenic bacterial cultures. The selection of Pt-complexes consisted of two Pt-chloro complexes (Pt(II)Cl_4_^2-^, Pt(IV)Cl_6_^2-^), the Pt-chemotherapy complexes cisplatin (*cis*-PtCl_2_[NH_3_]_2_) and carboplatin (*cis*-(Pt[NH_3_]_2_[1,1-cyclobutanedicarboxylato])), and a Pt-tetraamine complex (Pt(NH_3_)_4_Cl_2_). The biological platinum recovery was studied using three Gram-negative (G^-^) species (*Shewanella oneidensis* MR-1, *Cupriavidus metallidurans* CH34 and *Geobacter metallireducens*), each known for their ability to reduce metals, as shown for palladium [14, 20, 21], and extended with the Gram-positive (G^+^) species *Bacillus toyonensis* and the G^-^ species *Pseudomonas stutzeri*, both isolated from a wastewater treatment plant which processes metal streams.

### Influence of Pt-speciation on microbial recovery

The recovery of the five Pt-complexes was tested at pH 2 by using each of the cultures, in the presence of H_2_, formate or acetate as electron donor to study dissimilatory metal reduction, and without electron donor to evaluate sorption. The Pt-chloro complex Pt(II)Cl_4_^2-^ was recovered completely with H_2_ (**Table 1**) and a black microbial Pt suspension was formed (indicative for Pt reduction) [22]. The addition of formate or acetate as electron donor was less effective; 56–79% and 6–19% PtCl_4_^2-^ was recovered with formate and acetate, respectively (**S1 Table**). In experiments studying sorption, 6–25% of the dosed Pt was recovered on the biomass, however no color change was observed.

**Table 1:** An overview of the platinum recovery efficiencies (%) at pH 2 is given; the Pt recovery was investigated with and without (sorption control) the addition of H_2_-gas. The platinum recovery was studied using five different bacterial species and five Pt-complexes (n = 1). All recoveries were measured after 48 h, except for: * 68 h, ** 107 h and *** 168 h, and **** 320 h. The chemical reduction was studied for all Pt-species using H_2_-gas.

	Pt-species	Pt(II)Cl_4_^2-^		Pt(IV)Cl_6_^2-^		Pt(II)(NH_3_)_4_Cl_2_		Cisplatin		Carboplatin	
Culture		Sorption	H_2_	Sorption	H_2_	Sorption	H_2_	Sorption	H_2_	Sorption	H_2_
*S*. *oneidensis*		25	99	8	99***	0	0	8	99	3	6
*C*. *metallidurans*		24	99	8	99	0	0	3	99	1	3
*G*. *metallireducens*		6*	99	5	98**	-					
*B*. *toyonensis*		15	98	3	99****	0	0	0	5	7	1
*P*. *stutzeri*		18	99	2	99	0	0	8	10	10	9
**Chemical reduction**		-	99	-	0	-	0	-	99	-	0

The kinetics of the microbial reduction of Pt(IV)Cl_6_^2-^ were slower compared to Pt(II)Cl_4_^2-^; whereas almost complete recovery was observed in the case of H_2_ and 49–87% recovery with formate, no substantial Pt recovery was obtained using acetate (Table 1, S1 and **S2 Tables**). The sorption control showed a limited removal of 2–8%. Based on these Pt recovery efficiencies, hydrogen gas was preferred as sole electron donor for further experiments.

Cisplatin and carboplatin showed a very different behavior. Although both species sorbed on the biomass to a very little amount, only cisplatin was reduced successfully by the bacteria. Finally, the Pt-tetraamine complex was neither sorbed on any of the studied microbial species, nor reduced by these same species.

### Effect of the bacterial species on Pt recovery

Whereas all studied bacterial species recovered Pt(II)Cl_4_^2-^ quickly (within 2–4 h), with the G^+^ species *B*. *toyonensis* showing the slowest kinetics, none of them could recover Pt-tetraamine. Differences in recovery efficiency and kinetics were mainly observed for Pt(IV)Cl_6_^2-^ and cisplatin. *P*. *stutzeri* reduced PtCl_6_^2-^ remarkably quickly (< 24 h), whereas *S*. *oneidensis* and *B*. *toyonensis* were only able to recover the complex over an extended time period (≥ 1 week). A non-active biological control, i.e. heat-killed *Shewanella oneidensis* cells in the presence of H_2_-gas, removed 99% PtCl_4_^2-^ and 13% PtCl_6_^2-^, but at a slower rate (mainly between 24–48 h).

In the case of cisplatin, *S*. *oneidensis* and *C*. *metallidurans* could fully recover the complex, while *B*. *toyonensis* and *P*. *stutzeri* recovered at maximum 10%. In contrast, all bacterial species showed limited recovery of carboplatin.

To explore the recovery potential of the Pt-complexes under circumneutral conditions as a proxy for their fate in wastewater treatment plants or the environment, their recovery was also examined at pH 7 in the absence of an electron donor (**S1 Table**). In general, lower recovery efficiencies were noted under sorptive conditions compared to the dissimilatory reduction, showing the need for an electron donor to obtain full recovery. By using *S*. *oneidensis* and *C*. *metallidurans*, the platinum recovery at neutral pH was observed to be very similar to the sorptive removal at acidic pH. For *B*. *toyonensis* and *P*. *stutzeri*, a better sorption was noted under neutral conditions for mainly cisplatin, carboplatin, and PtCl_4_^2-^ on the long term (28–40% PtCl_4_^2-^ was recovered after 117–144 h) (**S1 Table**).

### Platinum speciation analysis

A characterization of the bacteria-metal interaction using X-ray absorption spectroscopy, was executed on the species *Shewanella oneidensis* MR-1, *Cupriavidus metallidurans* CH34 and *Geobacter metallireducens* removing the platinum chloro-complexes Pt(II)Cl_4_^2-^ and Pt(IV)Cl_6_^2-^. Anaerobically grown *S*. *oneidensis* was included in this speciation analysis for comparison but was not further investigated in this study.

**Fig 1:**
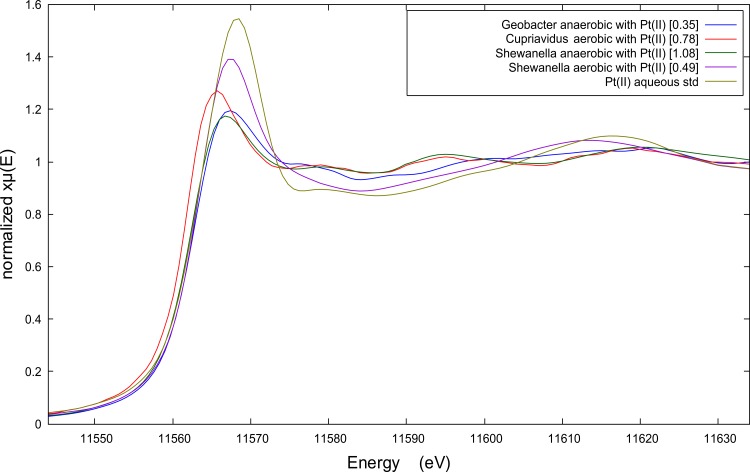
X-ray absorption near edge spectroscopy (XANES) spectra of biomass pellet samples after Pt(II)Cl42- recovery (100 mg L^-1^ Pt; 50 mg L^-1^ Pt in case of anaerobic *S*. *oneidensis*), by three bacterial species: *Geobacter metallireducens*, *Cupriavidus metallidurans* CH34 and *Shewanella oneidensis* MR-1.

The X-ray energy of peak fluorescence along with the overall shape of the Pt K-edge XANES spectra provides measures of the average oxidation state and bonding environment of Pt associated with the biomass. The peak fluorescence of all the spectra in **Fig 1**has shifted to lower energies relative to the Pt(II) aqueous spectrum indicating that the Pt associated with the biomass underwent reduction during the recovery process. The spectral patterns between peak fluorescence and 11 600 eV for Pt(II) recovered by aerobic *C*. *metallidurans* and anaerobic *S*. *oneidensis* are consistent with metallic Pt(0) particles [11], while the spectral patterns of anaerobic *G*. *metallireducens* and aerobic *S*. *oneidensis* are similar to the Pt(II) aqueous spectrum suggesting only partial reduction of the available Pt. The EXAFS spectra and Fourier Transforms (FTs) shown in the Supporting Information provide further evidence for these interpretations of the XANES spectra (see **S1 Fig**).

The XANES spectra also show how the level of Pt recovery (amount of Pt incorporated into the biomass) is correlated with each organism’s ability to reduce Pt(II) to metallic Pt(0) nanoparticles. Prior to normalization the edge jump in the XANES spectra reflects the number of Pt atoms fluorescing within the x-ray beam, and therefore, the height of the edge jump provides a measure of the relative amount of Pt per unit of biomass assuming equivalent biomass densities and sample thickness for all biomass samples. These assumptions are reasonable given that all underwent the same biomass separation method and were loaded in identical sample holders. The edge jump values (values shown in square brackets in **Fig 1**) increase as the Pt XANES spectra more closely resemble the XANES spectrum of metallic Pt(0) nanoparticles [11]. Without independent measures of Pt:biomass, the XANES edge jump only provides a relative measure of Pt recovery.

**Fig 2:**
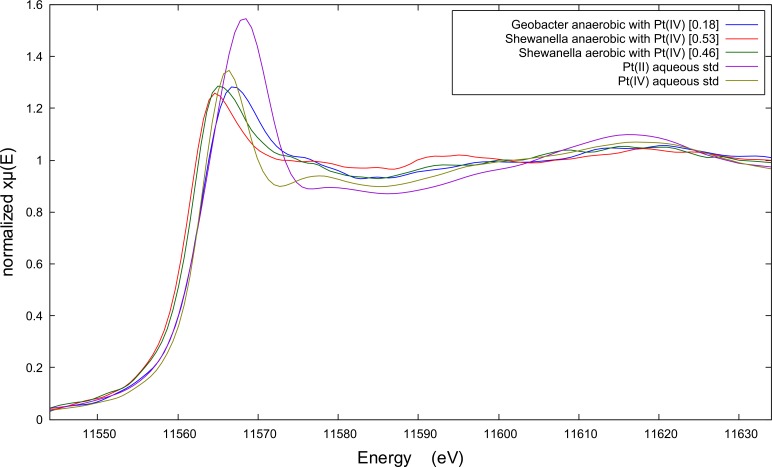
X-ray absorption near edge spectroscopy (XANES) spectra of biomass pellet samples after Pt(IV)Cl62- recovery (100 mg L^-1^ Pt; 50 mg L^-1^ Pt in case of anaerobic *Shewanella*), by two bacterial species: *Geobacter metallireducens* and *Shewanella oneidensis* MR-1.

All bacterial strains are less efficient at reducing and recovering aqueous Pt(IV) ions under anaerobic conditions according to the spectral edge jumps reported in **Fig 2**(see values in square brackets). *C*. *metallidurans* was below detection and therefore its spectrum is not shown. Anaerobic *G*. *metallireducens* and *S*. *oneidensis* reduced approximately half as much Pt relative to when Pt(II) is the starting aqueous species. It needs to be noted that the mismatch between the amount of Pt that was reduced to Pt(0) (XANES spectra) and the total amount of Pt that was recovered from solution (ICP data) corresponds with unreduced or partly reduced Pt. The energy of peak fluorescence in the XANES spectrum is a less reliable diagnostic given that the peak fluorescence of Pt(IV) aqueous standard occurs at a similar energy to reduced Pt(0) in biomass. However, the EXAFS spectra indicate that only the anaerobically grown *S*. *oneidensis* is capable of reducing significant amounts of Pt(IV) to Pt(0). The FT’s of these EXAFS spectra do suggest that a minor fraction of the Pt(IV) could have been reduced by anaerobic *G*. *metallireducens* and aerobic *S*. *oneidensis* (see **S2 Fig**).

### Platinum particle morphology

**Fig 3:**
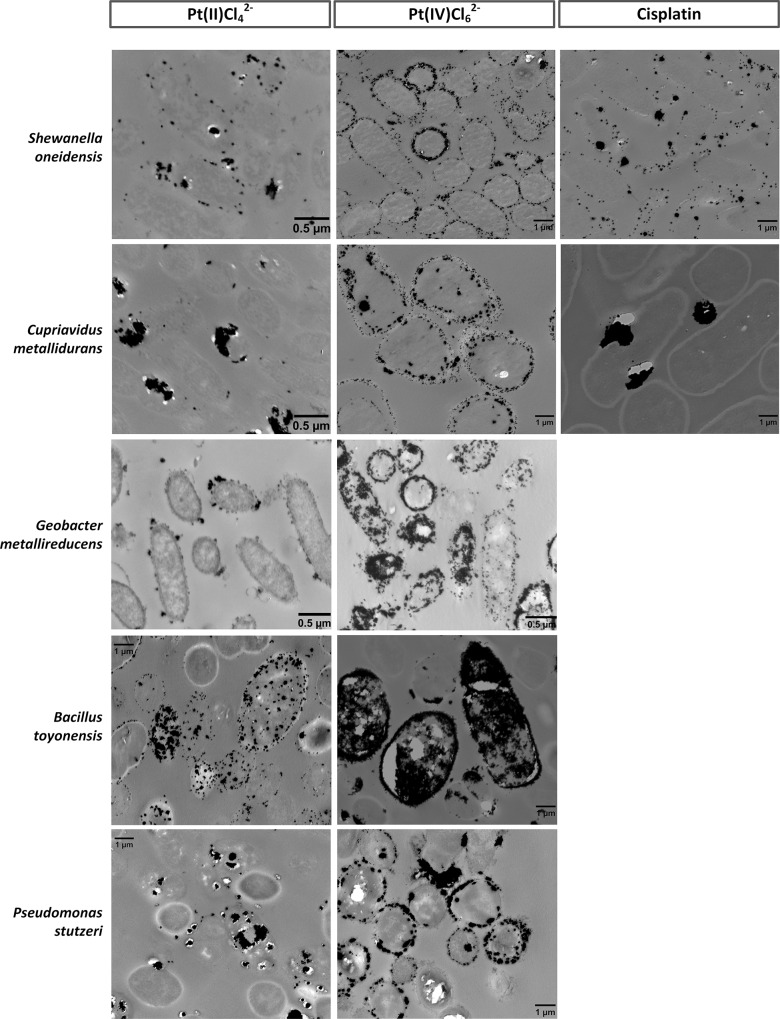
Transmission electron microscopy (TEM) images of thin sections of the five different bacterial species, loaded with platinum particles. The precipitation of platinum was induced by the presence of hydrogen gas. No Pt particles were observed during the recovery of cisplatin by *Bacillus toyonensis* and *Pseudomonas stutzeri*, while *Geobacter metallireducens* was not studied for this complex.

The characteristics of the Pt precipitates in and on the microbial biomass were studied using transmission electron microscopy (TEM). All bacterial suspensions that showed visible signs of Pt reduction were analyzed (*i*.*e*. black discoloration). The morphology of the platinum particles differed considerably according to the studied bacterial species and the recovered Pt-complex **(Fig 3**and **S3 Fig)**.

PtCl_6_^2-^ generally induced the formation of larger particles compared to PtCl_4_^2-^, as could be concluded from the particle size distributions (**S4 and S5 Figs**). For example, 24% (with formate) to 80% (with H_2_) of the total particle surface area was allocated to particles larger than 100 nm for the reduction of Pt(IV)Cl_6_^2-^ by *G*. *metallireducens*, compared to 15–23% for the reduction of Pt(II)Cl_4_^2-^ by the same bacterial species. Precipitated cisplatin also formed mainly larger particles (min. 71% of the particles were larger than 100 nm).

The Pt-speciation influenced the location of the formed precipitates as well, as observed for *B*. *toyonensis*. Whereas PtCl_4_^2-^ precipitated as dispersed, small intra- and extracellular particles, large Pt clusters completely filled the cells in the case of PtCl_6_^2-^ (**Fig 3**). Overall, both intra- and extracellular particles were observed for all three Pt-complexes, depending on the bacterial species. The increased importance of large particles in the particle surface area also corresponds to the increase in edge jump value for Pt(II)Cl_4_^2-^ (see **Fig 1**and **S4 Fig**).

*S*. *oneidensis* precipitated particles mainly on the cell wall and in the periplasmic space. Depending on the conditions (Pt-complex, electron donor), *C*. *metallidurans* tended to precipitate platinum into larger clusters, which can be observed in the case of cisplatin where very large clusters were located near the bacterial cells. Numerous small particles can be observed in the presence of *G*. *metallireducens*, which can cluster together to bigger particles such as in case of PtCl_6_^2-^. A similar clustering was observed after the reduction of PtCl_6_^2-^ by *B*. *toyonensis*; cells were completely filled with precipitated platinum. *P*. *stutzeri* reduced the Pt-chloro complexes into larger particles as well.

Next to the applied bacterial species and recovered Pt-complex, also the choice of electron donor influenced the precipitation of platinum. In general, larger particles were formed in the presence of H_2_-gas, compared to the presence of formate. Small uniformly dispersed particles were only observed in case of formate induced reduction (with *S*. *oneidensis* and *G*. *metallireducens*) (**S3 Fig**), which is in contrast with the palladium (Pd) study from De Windt et al. [20], that observed more small Pd particles in case H_2_ was used compared to formate.

Platinum precipitates formed in a previous study by *Shewanella algae* with Pt(IV) and lactate showed similarities with our results [13]. Pt particles of about 5 nm were observed in the periplasmic space, which is similar to the case of formate induced PtCl_6_^2-^ reduction by *S*. *oneidensis*, which resulted in particles of 4.8 nm (mean size), mainly formed in the periplasmic space and on the cell wall of the cells. In general, more intracellular Pt particles were formed in the case of the platinum(IV) chloro-complex, as was previously observed by Maes *et al*. when using halophilic mixed cultures [11].

### Membrane integrity of axenic cultures during platinum recovery

**Fig 4:**
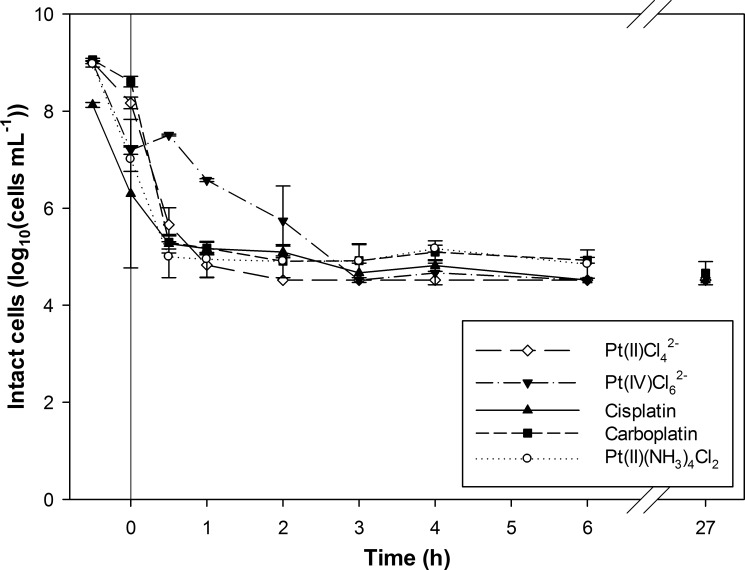
Membrane integrity of *Shewanella oneidensis* MR-1 cells during platinum recovery as a function of time. Five different Pt-complexes were dosed to investigate the effect of the Pt-speciation on the cell viability. The recovery experiment was initiated at t_0_ by the addition of 100 mg L^-1^ Pt and H_2_-gas as electron donor. The pH was initially set at pH 2.0.

The recovery of Pt-complexes and the prevalent conditions of the target wastewater will influence the viability of the present microorganisms and could affect their metal recovery potential. Furthermore, bacteria might behave differently towards the studied Pt-complexes according to the different intrinsic and chemical characteristics. Therefore, the cell viability of the axenic cultures was investigated during Pt-complex recovery experiments through flow cytometry based membrane integrity staining [23]. This staining makes it possible to distinguish cells with an intact cellular membrane, referred to as intact cells, from damaged cells. The influence of the Pt-speciation upon recovery was investigated using *S*. *oneidensis*, as the model organism **(Fig 4)**. The viability of the *Shewanella* culture decreased from approximately 10^9^ intact cells mL^-1^ to below the detection limit (33.3 x 10^3^ cells mL^-1^) within 2 hours after dosing of PtCl_4_^2-^. For PtCl_6_^2-^ and cisplatin, the number of intact cells decreased to the detection limit within 4 and 6 hours, respectively. The addition of the Pt-tetraamine complex and carboplatin lowered the amount of intact cells until approximately 10^5^ cells mL^-1^. These complexes were not reduced during the experiment, suggesting a damaging effect of the Pt reduction and the formation of intra- and extracellular precipitates. Furthermore, carboplatin is less toxic than cisplatin in chemotherapy treatments since it is more stable (due to a more stable leaving group), which might explain the more limited interaction in this study as well [24]. Although this study showed a slower decrease in intact cells in the case of PtCl_4_^2-^ compared to PtCl_6_^2-^ (based on the *S*. *oneidensis* culture), PtCl_6_^2-^ was previously found to be more toxic to *C*. *metallidurans* than PtCl_4_^2-^; minimal inhibitory concentrations of 39 mg L^-1^ and 3.4 mg L^-1^ were determined for respectively the Pt(II) and Pt(IV)-chloro complex [25]. In this study, the cell viability was however mainly linked to the precipitation of Pt particles instead of the intrinsic toxicity of the metal salts.

**Fig 5:**
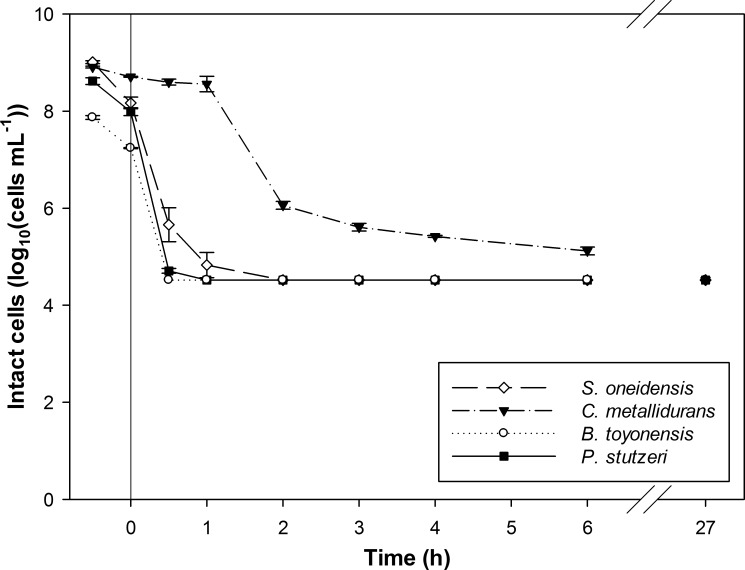
The effect of the addition of Pt(II)Cl42- on the membrane integrity of different bacterial cells during platinum recovery as a function of time. Four different bacterial cultures were studied; *Shewanella oneidensis* MR-1, *Cupriavidus metallidurans* CH34, *Bacillus toyonensis* and *Pseudomonas stutzeri*. The recovery experiment was started at t_0_ by the addition of 100 mg L^-1^ Pt and H_2_-gas as electron donor. The pH was initially set at pH 2.0.

The PtCl_4_^2—^complex was selected as a toxic and industrially relevant Pt-complex to study the vulnerability of the different bacterial organisms towards Pt-complex toxicity **(Fig 5)**. Among the screened cultures, differences could be observed. The decrease in intact cells was almost identical for *B*. *toyonensis* and *P*. *stutzeri*; the detection limit was reached within 1 hour, indicating a severe damaging effect during Pt recovery. *C*. *metallidurans* appeared to be the most resistant species; 1.2 x 10^6^ intact cells mL^-1^ were still measured after 2 hours and 1.3 x 10^5^ intact cells mL^-1^ after 6 hours. This might be explained by the presence of metal resistance gene clusters in this species, enabling cell detoxification, as was demonstrated before for precious metals gold and silver [26, 27]. Still, the amount of intact cells of all bacterial suspensions decreased finally until the detection level (< 27 hours after the initiation of the experiment). Overall, the affected viability of the cultures will have been caused by the combined effect of (1) the acidic pH, (2) the exposure to the Pt-complexes, as an increased cell membrane permeability by Pt(IV) ions was observed before [22] and (3) the precipitation of Pt particles, previously shown for an *Acinetobacter* species [28]. The substantial effect of the acidic pH on the viability is shown for the *S*. *oneidensis* suspension containing cisplatin; 3.7 x 10^8^ intact cells mL^-1^ were still detected after 6 h when working at pH 5, while only 3.3 x 10^4^ intact cells mL^-1^ were measured at pH 2 (*i*.*e*. limit of detection). Next to metal toxicity, the pH of the metal containing wastewaters will be another important challenge to obtain an effective biological metal recovery.

### Interaction mechanisms between bacteria and Pt-species

The bacterial interaction in these biorecovery processes is believed to consist of two concomitant steps; (1) the initial sorption of Pt to the cell, which is followed by (2) the microbial reduction of the sorbed Pt-molecules [29, 30].

Differences observed during the first sorptive step might be partly related to the different cell wall structure of G^+^ and G^—^species. In general, the recovery of deprotonated complexes is favored by a low pH as functional groups on the bacterial surface become protonated at low pH [31]. Furthermore, the speciation and valence of Pt-complexes is highly dependent on the pH and chloride concentration. Chloro, hydroxyl or hydrated complexes can be formed depending on the conditions, characterized by a complicated chemistry [32–35]. For example, the Pt-chloro complexes PtCl_4_^2-^ and PtCl_6_^2-^, which are important species under acidic and saline conditions, are characterized by a negative charge at low pH [36]. This enables electrostatic interactions with positively charged binding sites such as amine groups and presumably results in effective recovery [12, 36]. The observed slower recovery and precipitation of Pt(IV) can be explained by the sequential transformation of Pt(IV) through Pt(II) to Pt(0) [37]. Additionally, PtCl_6_^2-^ is expected to be more difficult to reduce, based on the slightly lower standard reduction potential of this Pt-complex: E^0^(Pt(IV)Cl_6_^2-^ ⇔ PtCl_4_^2-^) = + 0.726 V vs. SHE compared to E^0^(PtCl_4_^2-^) = + 0.758 V vs. SHE [38]. The first reduction step of Pt(IV) to Pt(II) will have slowed down and limited the platinum recovery performance. Riddin et al. [37] proposed a dislocated two-step reduction of Pt(IV) by sulphate-reducing bacteria, in which Pt(IV) was reduced to Pt(II) by a cytoplasmic hydrogenase and Pt(II) further to Pt(0) by a periplasmic hydrogenase.

Furthermore, none of the bacterial species was able to recover the Pt-tetraamine complex. Since any sorption was lacking, the neutral complex Pt(NH_3_)_4_Cl_2_ will probably have formed, preventing any interaction with the bacterial surface and making a biological treatment ineffective. The uncharged cisplatin partitions (partially) into the hydrated complexes *cis-*[PtCl(NH_3_)_2_(H_2_O)]^+^ and *cis-*[Pt(NH_3_)_2_(H_2_O)_2_]^2+^ when low chloride concentrations are present [33]. The formation of these positively charged Pt-complexes might explain the limited sorption by protonated functional groups at low pH. Still, complete recovery was possible at low pH in the presence of H_2_-gas. The limited sorption of, for example, a residual amount of the uncharged mother compound can thus be sufficient to induce the full reduction of this Pt-complex, as long as a capable microbial species and an electron donor are present. The second chemotherapy complex carboplatin has been observed to mainly remain stable in wastewater and was characterized in our study by a limited recovery under all tested conditions [39]. The formation of Pt precipitates was only observed for cisplatin and not for carboplatin. The sorptive recovery of both chemotherapy complexes was studied before by using activated sludge, revealing the least sorption for carboplatin (70% vs. 96% for cisplatin) [32].

Different mechanisms might be responsible for the recovery potential of the studied bacterial species. The recovery of platinum was investigated before using the related marine species *Shewanella algae*, which was able to recover 90% PtCl_6_^2-^ within 1 hour using lactate as electron donor (C_0_ = 200 mg L^-1^ Pt at pH 7) [13]. To our knowledge, the recovery of platinum by the anaerobic species *Geobacter metallireducens* has not been studied yet, but the reduction of palladium was demonstrated recently by the related *Geobacter sulfurreducens* [21, 40]. The dissimilatory metal reducing bacteria *Shewanella* and *Geobacter* were found to use different hydrogenases and cytochromes, being present in the outer membrane, periplasm or cytoplasm, to transfer electrons to reduce the metals [40, 41]. The heavy metal-resistant and metallophilic species *Cupriavidus metallidurans* is well-studied for the reduction and precipitation of palladium and gold, initiated by the expression of different metal resistance genes [26, 42, 43]. Gauthier et al. [14] demonstrated the recovery of platinum and palladium by *Cupriavidus metallidurans* and *Cupriavidus necator* species in the presence of hydrogen gas; 70–74% Pt and 96–100% Pd were recovered from a mixed metal acidic leachate (pH 1.4; 24 h). The *Pseudomonas stutzeri* species has been shown to reduce selenate and selenite and to produce silver nanoparticles [44, 45]. It is hypothesized that siderophores, produced by the *Pseudomonas* species, are involved in a detoxification strategy of the species, by extracellularly complexing and reducing various metals [46]. The only studied G^+^ species, *Bacillus toyonensis*, has not been utilized yet in metal recovery studies, although *Bacillus* species have been shown to reduce palladium [47]. The recovery potential of these axenic cultures should be further explored under real stream conditions.

## Supporting Information

S1 Fig **(A) Extended X-ray Absorption Fine Structure (EXAFS) spectra and their (B) Fourier Transforms (FT) of biomass pellet samples after Pt(II)Cl**_**4**_^**2-**^ **recovery by three bacterial species:** *Geobacter metallireducens*, *Cupriavidus metallidurans* CH34 and *Shewanella oneidensis* MR-1.
(TIF)

S2 Fig **(A) Extended X-ray Absorption Fine Structure (EXAFS) spectra and their (B) Fourier Transforms (FT) of biomass pellet samples after Pt(IV)Cl**_**6**_^**2-**^ **recovery by two bacterial species:** *Geobacter metallireducens* and *Shewanella oneidensis* MR-1.
(TIF)

S3 Fig Transmission electron microscopy (TEM) images of thin sections of *Shewanella oneidensis* MR-1, *Cupriavidus metallidurans* CH34 and *Geobacter metallireducens* cells respectively, loaded with platinum particles. The precipitation of platinum was induced by the presence of formate as electron donor.
(TIF)

S4 Fig Platinum particle distributions of platinum nanoparticles (nm) formed by five different bacterial cultures (based on total particle surface area). The different Pt-complexes were precipitated in the presence of hydrogen gas. No Pt particles were observed during the recovery of cisplatin by *Bacillus toyonensis* and *Pseudomonas stutzeri*, while *Geobacter metallireducens* was not studied for this complex.
(TIF)

S5 Fig Platinum particle distributions of platinum nanoparticles (nm) (based on total particle surface area), formed by the bacterial species *Shewanella oneidensis* MR-1, *Cupriavidus metallidurans* CH34 and *Geobacter metallireducens*. The Pt(II)Cl_4_^2-^ and Pt(IV)Cl_6_^2-^ complexes were precipitated in the presence of formate as electron donor.
(TIF)

S1 Table Platinum recovery efficiencies (%) are given under different recovery conditions; the sorption of all Pt-complexes was studied at pH 7 while the reduction (pH 2; with formate or acetate) was only studied for Pt(II)Cl42- and Pt(IV)Cl62- using the *Shewanella oneidensis* MR-1, *Cupriavidus metallidurans* CH34 and *Geobacter metallireducens* species (n = 1). All recovery efficiencies were measured after 48 h, except for: * 68 h, ** 107 h, *** 117 h and **** 144 h. The chemical reduction using formate was studied for Pt(II)Cl_4_^2-^ and Pt(IV)Cl_6_^2-^.
(TIF)

S2 Table Additional platinum recovery efficiencies (%) for Pt(IV)Cl62-, measured after 48 h, for *Shewanella oneidensis* MR-1, *Geobacter metallireducens* and *Bacillus toyonensis* species with different electron donors (formate, acetate or H_2_; pH 2) (n = 1). (TIF)
